# Nuclear hormone receptors in podocytes

**DOI:** 10.1186/2045-3701-2-33

**Published:** 2012-09-20

**Authors:** Simran Khurana, Leslie A Bruggeman, Hung-Ying Kao

**Affiliations:** 1 Department of Biochemistry, School of Medicine, Case Western Reserve University (CWRU) and the Comprehensive Cancer Center of CWRU, 10900 Euclid Avenue, Cleveland, Ohio 44106, USA; 2 Rammelkamp Center for Education and Research and Department of Medicine, MetroHealth Medical Center, Case Western Reserve University School of Medicine, Cleveland, Ohio, USA

**Keywords:** Glucocorticoid receptor, Mineralocorticoid receptor, Podocyte injury, Proteinuria, Focal segmental glomerulosclerosis (FSGS), Diabetic and non-diabetic nephropathy

## Abstract

Nuclear receptors are a family of ligand-activated, DNA sequence-specific transcription factors that regulate various aspects of animal development, cell proliferation, differentiation, and homeostasis. The physiological roles of nuclear receptors and their ligands have been intensively studied in cancer and metabolic syndrome. However, their role in kidney diseases is still evolving, despite their ligands being used clinically to treat renal diseases for decades. This review will discuss the progress of our understanding of the role of nuclear receptors and their ligands in kidney physiology with emphasis on their roles in treating glomerular disorders and podocyte injury repair responses.

## Overview

Nuclear hormone receptors (NRs) belong to family of sequence specific and ligand activated transcription factors that both positively and negatively regulate gene expression and are involved in many developmental, cell survival, and endocrine functions in metabolism [1,2]. Consequently, aberrant NR signaling can lead to various reproductive, proliferative, and metabolic diseases. The ability of small molecule hormones to regulate NR activity make them excellent pharmaceutical targets; for example, retinoic acid (a ligand for retinoic acid receptor alpha, RARα), the synthetic antagonist tamoxifen (a ligand for estrogen receptor alpha, ERα), dexamethasone (a ligand for glucocorticoid receptor alpha, GRα) or thiazolidinediones (ligands for peroxisome proliferator-activated receptor gamma, PPARγ) are used in acute promyelocytic leukemia, ERα-positive breast cancer, inflammatory disorders and type II diabetes, respectively [3]. This family of hormones also plays important roles in the kidney development, in adult renal homeostasis and in disease responses. The kidney functions in the physiologic maintenance of acid–base and salt-water balance, and in removing toxins and metabolic waste while preserving nutrients in the bloodstream. The latter filtration function is largely mediated by the glomerular podocyte, a highly differentiated kidney cell that opposes the exterior of fenestrated capillaries in the renal glomerulus. Loss or injury of podocytes results in impaired blood filtration and causes many common renal diseases characterized by nephrotic syndrome. In this review, we will discuss the roles of NRs in normal podocyte development and in glomerular diseases and their physiological hormones and synthetic ligands as potential treatments for nephrotic syndrome.

## Nuclear hormone receptors: classification and functional domains

In humans, there are 48 NRs that can be broadly classified into four subfamilies based on their ligand binding, DNA binding and dimerization properties (Table 1). NRs bind to their DNA response elements either as monomers, dimers, or heterodimers. Class I receptors bind to DNA inverted repeats as homodimers, and include the estrogen receptor (ER), glucocorticoid receptor (GR), mineralocorticoid receptor (MR), progesterone receptor (PR) and androgen receptors (AR). Class II receptors bind to DNA direct repeats and heterodimerize with retinoid X receptors (RXR), and include the thyroid hormone receptors (TR), retinoic acid receptors (RAR) and retinoic X receptors (RXR), peroxisome proliferator-activated receptors (PPAR), vitamin D3 receptors (VDR), and Liver X receptors (LXR). Receptors belonging to class III are known as orphan receptors since their natural ligands have not been identified. These NRs bind to the DNA direct repeats as homodimers. Class IV receptors are also orphan receptors but bind to DNA as monomers [3].

**Table 1:** Classification of nuclear hormone receptors

**Class**	**Nuclear Receptor**	**Abbreviation**	**Hormones and Synthetic Ligands**
I	Androgen receptor	AR	testosterone, flutamide
	Estrogen receptor, alpha and beta	ERα, β	estrogens, tamoxifen, raloxifene
	Glucocorticoid receptor	GR	glucocorticoidl, dexamethasone, RU486
	Mineralocorticoid receptor	MR	aldosterone, spirolactone
	Progesterone receptor	PR	progesterone, medroxyprogesterone acetate, RU486
II	Constitutive and rostane receptor	CAR	androstane
	Farnesoid X receptor	FXR	bile acids, Fexaramine
	Liver X receptor, alpha and beta	LXRα, β	oxysterols, T0901317, GW3965
	Peroxisome proliferator-activated receptor alpha	PPARα	fibrates
	Peroxisome proliferator-activated receptor beta/delta	PPARβ/δ	fatty acids
	Peroxisome proliferator-activated receptor gamma	PPARγ	prostaglandins
	Pregnane X receptor	PXR	xenobiotics
	Retinoid A receptor, alpha, beta and gamma	RARα, β, γ	all-trans retinoic acid
	Thyroid hormone receptor, alpha and beta	TRα, β, γ	thyroid hormone
	Vitamin D receptor	VDR	vitamin D
III	Chicken ovalbumin upstream promoter-transcription factor I	COUP-TFI	n/a
	Chicken ovalbumin upstream promoter-transcription factor II	COUP-TFII	n/a
	V-erbA-related receptor	EAR-2	n/a
	Germ cell nuclear factor	GCNF	n/a
	Hepatocyte nuclear factor-4, alpha and gamma	HNF4α, γ	Fatty acids
	Photoreceptor cell-specific nuclear receptor	PNR	9-cis retinoic acids
	Retinoid X receptor, alpha, beta and gamma	RXRα, β, γ	9-cis retinoic acids
	Testicular receptor 2	TR2	n/a
	Testicular receptor 4	TR4	n/a
	Homologue of the Drosophila tailless gene	TLX	n/a
IV	Estrogen-related receptor alpha, beta and gamma	ERRα, β, γ	9-cis retinoic acid
	Liver receptor homolog-1	LRH-1	phosphatidylinositols
	Nerve Growth factor IB	NGFIB	n/a
	Neuron-derived orphan receptor 1	NOR1	N/As
	Nuclear receptor related 1	NURR1	N/A
	Rev-ErbA, alpha and beta	Rev-Erbα, β	heme
	RAR-related orphan receptor, alpha, beta and gamma	RORα, β, γ	cholesterol, all-trans retinoic acids
	Steroidogenic factor 1	SF1	phosphatidylinositols
	Dosage-sensitive sex reversal, adrenal hypoplasia critical region, on chromosome X, gene	DAX	n/a
	Small heterodimer partner	SHP	n/a

**Figure 1:**
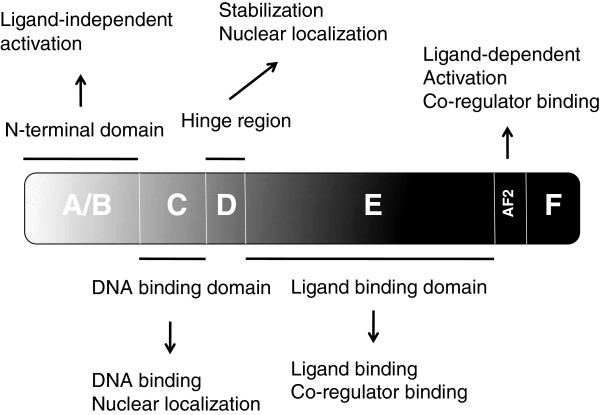
** A schematic representation of a nuclear receptor: Nuclear receptors consist of four domains (A-F): The N-terminal ligand-independent transactivation domain (A/B), the DNA binding domain (C), hinge region (D), and C-terminal E/F domain including LBD and ligand dependent transactivation domain.** Functions of specific domains are indicated in the text boxes.

All NRs are evolutionarily related [3] and have a common modular structure consisting of four domains (Figure 1). Among these domains, the DNA binding domain (region C) and the ligand binding domain (region E) are the most highly conserved, whereas the N-terminal A/B domain and the D region are comparatively less well conserved [3]. The A/B domain harbors an activation domain that stimulates transcription in a ligand-independent manner. In different NRs, both the length and the sequence of the A/B region are variable. The central C-domain of NRs is the evolutionarily conserved DNA binding domain. NRs bind DNA at highly specific nucleotide sequence motifs of 5–10 base pairs generally known as hormone response elements (HREs) [4-7]. The DNA binding domain consists of two cysteine-rich zinc finger motifs, two α-helices, and a C-terminal extension, and plays important roles in both nuclear localization and in the interaction with other transcription factors [3]. The D region serves as a linker between the DNA binding domain and the ligand-binding domain and contains a nuclear localization signal. The ligand binding domain is contained in the C-terminal E region and harbors four functionally interconnected regions including the ligand-binding pocket, a dimerization surface and a transcriptional co-regulator binding surface which participates in protein-protein interactions with other transcription factors, and an activation helix known as AF-2, which mediates ligand dependent transactivation [8].

## Mechanism of transcriptional regulation by NRs

NRs regulate transcription through the recruitment of accessory proteins known as co-regulators (coactivators and corepressors) that dictate the transcriptional activity of the receptors. In the absence of ligand, NRs including RAR, TR, and antagonist-bound steroid receptors, recruit corepressors such as nuclear receptor corepressor (NCoR) and silencing mediator for retinoid and thyroid hormone receptors (SMRT) to inhibit transcription initiation [9,10]. Unliganded steroid hormone receptors such as GR, MR and ER do not normally interact with NCoR or SMRT, but interact strongly with these corepressors in the presence of antagonists [11-13]. Chromatin immunoprecipitation assays have shown that N-CoR and SMRT complexes are recruited to NR targeted promoters [10]. Both NCoR and SMRT contain a region at their C-termini that specifically binds to a hydrophobic groove in the surface of the ligand-binding domain of unliganded NRs. NCoR and SMRT interact with unliganded receptors through a conserved helical motif (I/L) XX (I/V) I (L = leucine, I = isoleucine, V = valine and X = any amino acid) [14-16]. Both NCoR and SMRT do not possess intrinsic enzymatic activity; however, they recruit other proteins containing histone deacetylase (HDACs) and methyltransferase (SUV39H1) activity [17,18]. HDACs repress transcription by deacetylating lysine residues on the N-terminal tails of histone proteins. This condenses the chromatin, which in turn restricts access of the basic transcriptional machinery to the target promoter.

**Figure 2:**
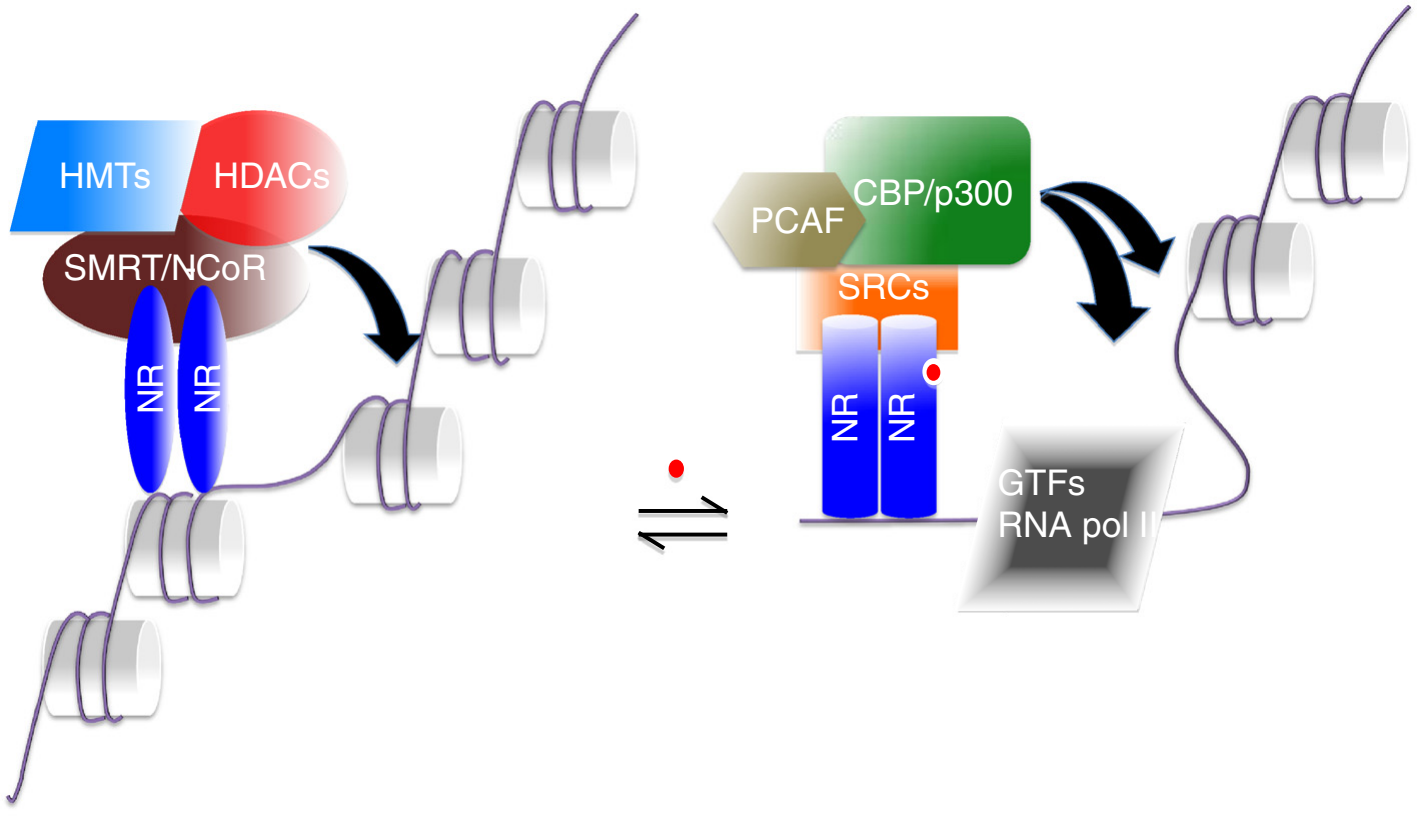
** Ligand-dependent conformational change and transactivation of a nuclear receptor.** In the absence of ligand, nuclear receptors are associated with corepressor complexes such as SMRT, HDACs and histone methyltransferases (HMTs) and inhibit transcription by keeping the chromatin tightly bound around the promoter. Ligand binding induces a conformational change in the structure of nuclear receptors which exchanges the corepressors with coactivators. The coactivators including CBP/p300, PCAF and SRCs loosen chromatin by acetylating histone tails. Acetylation of histone tails opens up the chromatin which in turn allows basal transcriptional machinery to target promoters.

Crytallographic studies have shown that ligand binding triggers a conformational change in the ligand-binding domain of the receptor [19-23]. This conformational change is accompanied by release of the corepressor complexes and recruitment of the coactivator complexes containing histone acetyltransferase and methyltransferase activity [10] (Figure 2). Binding of these coactivators allows the subsequent recruitment of RNA polymerase II and general transcription machinery to a targeted promoter [24], thereby stimulating transcription. Structurally, helix 12 of the AF-2 region of the ligand-binding domain plays an important role in the recruitment of coactivators. Upon ligand binding, there is a reorientation of helix 12 which results in the formation of a hydrophobic groove that accommodates coactivator binding. Thus, this co-regulator exchange ultimately controls transcription through the steric opening or closing of the local chromatin structure through the modification of histone tails.

## Significance of the LXXLL motif in coactivators in NR-mediated transcription

Coactivators interact with NRs through the highly conserved NR interaction domain known as the NR box. The NR interaction domain consists of a short α-helical LXXLL motif. The number of LXXLL motifs varies among different coactivators and also accounts for the preferential binding of some coactivators to a specific NR [25-29]. Among the coactivators discovered so far, the SRC (steroid receptor coactivator) family of proteins, CBP (cAMP response element-binding protein) and p300 are known for their ability to interact with and co-activate NRs [30-33]. The SRC family of coactivator consists of three family members: SRC1, the first identified nuclear receptor coactivator (also known as p160-1 and N-CoA1), SRC-2 (TIF-2, GRIP1, and N-CoA2) and SRC3 (also known as P/CIP, ACTR, AIB1, RAC3, and TRAM1) [34]. All three SRC family members share a common domain structure and have three equally spaced conserved LXXLL motifs to interact with NRs. Note that most coactivators including PCAF (p300/CBP-associated factor) independently interact with NRs and with each other. Chromatin immunoprecipitation experiments have demonstrated that these coactivators are recruited to NR targeted promoters in a sequential, cyclic manner [35]. All together, these observations suggest that SRC coactivators function by recruiting chromatin modifying enzymes to the liganded receptors on the HREs. PCAF and p300/CBP harbor potent HAT activity, while the C-termini of SRC-1 and SRC-3 exhibit weak HAT activity [3].

**Figure 3:**
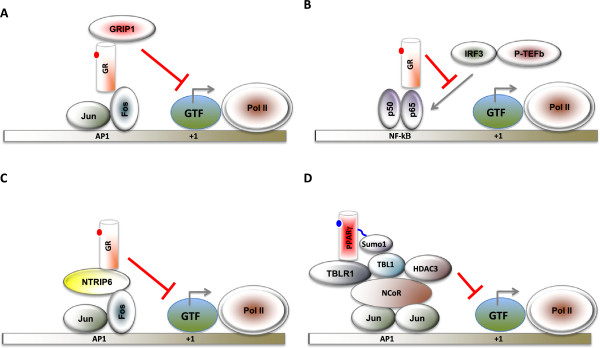
**Mechanisms underlying NR-mediated transrepression. *A***, Liganded GR binds to Fos subunit of activator protein 1 (AP-1) and represses a subset of AP-1-dependent genes through GR interacting protein 1(GRIP-1). ***B***. Liganded GR binds to p65 subunit of nuclear factor-κB (NF-κB) and prevents the binding of interferon regulatory factor 3 (IRF3) or positive transcription elongation factor b (P-TEFb) to the promoter of some NF-κB target genes. ***C***, Liganded GR binds to Fos subunit of AP-1 and represses a subset of AP-1 dependent genes through nuclear thyroid receptor interactor 6 (NTRIP6). ***D***, Liganded PPARγ (or LXR) is posttranslationally modified by Sumo1 (or Sumo2) conjugation, which facilitates an interaction with nuclear receptor corepressor (NCoR) complex to inhibit the recruitment of ubiquitin-conjugating enzymes and 19S proteasome components (not shown) required for the degradation of NCoR. Transcription start site is shown as +1. GTF refers to the general transcription factors. Pol II refers to RNA polymerase II. This figure is adopted from Glass and Saijo (42).

Some NRs are capable of eliciting ligand-dependent transcriptional repression activity [36]. Liganded GR and TR can repress gene expression through negative response elements [37,38]. In addition, NRs such as GRs, PPARs, LXR, VDR and RAR repress NF-κB and AP-1 target gene expression in an agonist-dependent manner [39] (Figure 3). The mechanisms underlying this transrepression activity include a) competition with coactivators [40,41] b) disruption of the recruitment of positive acting complexes and c) sumoylation-dependent recruitment of corepressor complexes to AP-1 and NF-κB targeted promoters [42]. It should be noted that many of these AP-1 and NF-κB target genes are key mediators of the inflammatory response.

## Role of NRs in glomeruli and related diseases

While the physiological roles of NRs and their ligands have been intensively studied in cancer and metabolic syndrome X, understanding their roles in kidney development and podocytes is still evolving. It has been a longstanding clinical practice to use NR ligands to treat kidney diseases, especially nephrotic syndrome and diseases that damage the glomerulus and podocyte, despite the lack of a clear understanding of their mechanism of action. Recent studies in multiple experimental models of renal diseases have begun to investigate the direct and indirect effects of NR in renal cells to better utilize NR ligands as therapeutic agents in common diseases such as focal segmental glomerulosclerosis (FSGS).

## Podocytes – terminally differentiated cells critical for kidney filtration function

One of the key functions of the kidney is to filter the blood, removing catabolic byproducts that can become toxic if not eliminated. The filtration apparatus of the kidney is the glomerulus, a tuft of capillaries, consisting of three components: the fenestrated glomerular endothelium, the glomerular basement membrane, and a visceral epithelium also known as the podocyte. Podocytes cover the exterior of the capillary and attach to the outer layer of the glomerular basement membrane. They form novel marcromolecular structures that function like a molecular sieve, allowing high volume fluid flow while preventing passage of blood cells and large serum proteins such as albumin from entering the urine. Podocytes also contribute significantly to the formation of the glomerular basement membrane and the integrity of the vascular endothelium. Thus, podocyte damage is a hallmark of nephrotic syndrome characterized by severe proteinuria (protein in the urine) and hypoalbuminemia (low levels of blood albumin).

**Figure 4:**
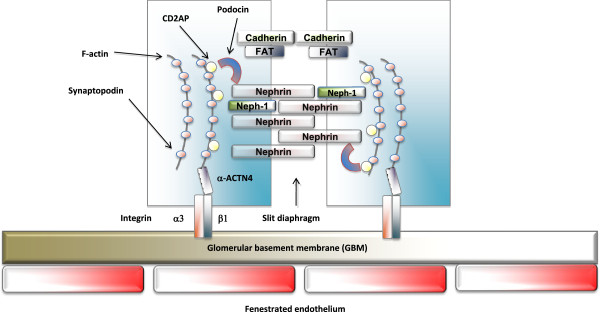
**The structure of podocyte.** The glomerular capillary wall consists of a fenestrated endothelium, a basement membrane and podocyte foot processes. The foot processes form the filtration slits and are spanned by slit diaphragms. The slit diaphragm is composed of the extracellular domains of a number of unique transmembrane proteins such as Nephrin, Neph-1, P-cadherins and FAT.

Podocytes are highly specialized terminally differentiated cells that extend numerous lamellipodia that branch into primary and secondary processes, which further ramify into smaller processes known as foot processes [43]. The latter are composed of highly ordered parallel contractile actin filament bundles [44,45]. Foot processes from neighboring cells interdigitate and are connected by a modified adherent junction called slit diaphragms that span a 30–50 nm wide intercellular space that provides for the passage of fluid. The slit diaphragm of podocytes is composed of the extracellular domains of a number of transmembrane proteins such as nephrin, Neph-1, P-cadherins and FAT [45] (Figure 4). The ability of podocytes to act as a filtration barrier depends on the integrity of the slit diaphragm [43]. Recent studies have indicated that the components of foot processes not only serve as a structural barrier; they also respond to and mediate extracellular signaling events and are indispensable for proper physiological responses of the podocyte to the environment [46].

## Focal segmental glomerulosclerosis

Focal segmental glomerulosclerosis is common pathological condition consisting of local and sectional degeneration and scarring within the glomerular tuft. It occurs in many inherited and acquired kidney disease, and is frequently a consequence of direct podocyte injury. Upon injury, the podocyte responds with cytoskeletal reorganization and ultimately the foot processes become disorganized. At the molecular level, podocyte injury is associated with 1) increased reactive oxygen species (ROS) and endoplamic reticulum (ER) stress, 2) aberrant activation of mammalian target of rapamycin (mTOR), Wnt and transforming growth factor beta (TGF-β) signaling, 3) activation of small GTPase RhoA and 4) decreased expression of slit diaphragm components such as nephrin [47]. Any type of oxidative or inflammatory stress leading to effacement of podocyte foot processes further deteriorates the filtration barrier and overall kidney function, and eventually results in renal failure. Genetic studies have identified mutations in many known podocyte structural proteins such as the slit diaphragm components nephrin, CD2 associated protein (CD2AP), transient receptor potential cation channel, subfamily C, member 6 (TRPC6) and podocin or in proteins regulating actin dynamics including alpha actinin 4 (ACTN4) and inverted formin 2 (INF2) as being tightly linked to FSGS. Animal studies confirm that knock-in of disease-associated mutations or podocyte-specific gene deletions leads to FSGS. These studies highlight the important role of the slit diaphragm and actin architecture in the integrity of podocytes. A reverse correlation between the degree of proteinuria and nephrin expression levels has also been documented [48-50]. Furthermore, mutations in the *ACTN4* and synaptopodin promoters have been found in patients and these mutations are associated with reduced promoter activity [51], suggesting that loss of nephrin, ACTN4 or synaptopodin expression may contribute to FSGS. These findings further imply suggest that mis-regulation of transcriptional networks controlling slit diaphragm component gene expression can contribute to podocyte disorders. Alternatively, focal segmental glomerulosclerosis can be caused secondarily from metabolic or immunologic dysfunction that also leads to podocyte injury and proteinuria [52,53].

## NRs in podocyte pathophysiology

To date, there are no known mutations in NRs that are linked to familial forms of focal segmental glomerulosclerosis and nephrotic syndrome. However, clinical evidence and mouse genetic studies have implicated several NRs in contributing to podocyte development and disease (Table 2). Among the known NRs, the mineralocorticoid receptor (MR) and its agonists have been strongly associated with proteinuria [54,55]. Recent studies from animals and cultured human or mouse podocytes indicate that synthetic hormones for class I and II NRs including estradiol, glucocorticoid, retinoid, pioglitazone, vitamin D3 and WY-14643 protect or rescue podocytes from experimental injury [56-60]. Post treatment of injured podocytes with ligands for the above-mentioned NRs restores cytoskeletal architecture and enhances expression of nephrin. A unified theme derived from clinical, animal and cell culture studies suggest that NRs, except MR, elicit renoprotective activity by inhibiting apoptosis, by acting as antioxidants and by enhancing/restoring nephrin expression.

**Table 2:** A summary of the physiological function of nuclear hormone receptors in podocytes

**Nuclear receptor**	**Role in kidney function/disease**	**Reference**
Estrogen receptor	renoprotective blocks podocyte apoptosis	[61-64]
Glucocorticoid receptor	well-known renoprotective functions of GR ligands	[56,65-67]
Mineralocorticoid receptor	renoprotective when suppressed	[68-74]
Peroxisome proliferator-activated receptor-α	renoprotective enhances Nephrin expression	[75-78]
Peroxisome proliferator-activated receptor-γ	renoprotective blocks podocyte apoptosis blocks podocyte hypertrophy/proliferation	[79-82]
Retinoic acid receptor-α	renoprotective enhances podocyte differentiation	[83-90]
Vitamin D receptor	reduces renal inflammation enhances Nephrin expression	[91-95]

## Class I NRs

Class I NRs including MR, GR, ER, PR and AR bind to the DNA inverted repeats (IRs) as homodimers (Table 1). Among these, MR and GR are the best studied in the kidney due to the early cloning of their genes and discovery of their physiological ligands. Furthermore, recently the physiological importance of ER in kidney pathophysiology has emerged.

## MR (mineroglucocorticoid receptor)

MR function in the kidney has been studied extensively focusing on its roles in controlling blood pressure and salt and water balance, functions primarily associated with the renal tubule not glomerulus. Consequently, the renally important MR ligand, aldosterone, is well-known to be involved in renal disease and pathology, and MR antagonists are used extensively in treating hypertension [68]. MR is normally expressed in many cells of the kidney, not just tubular epithelia, but also glomerular mesangial cells and podocytes [69,70,96]. Studies in various animal models have therefore also linked MR function to mesangial cell proliferation and podocyte injury and proteinuria [69,97-99]. Recent studies have identified podocytes as a direct target of aldosterone through the MR [55,69,71]. MR blockade with eplerenone (an antagonist of MR) reduces podocyte injury and proteinuria and induces podocin and nephrin expression in podocyte in type 1 and type 2 diabetic animal models [72,73,100]. By contrast, chronic infusion of aldosterone induces hypertension with massive proteinuria and glomerular podocyte injury in uninephrectomized (surgical excision of one kidney) rats [69]. Furthermore, the induced podocyte injury is associated with significantly reduced nephrin and podocin expression [101]. In addition, treatment with the MR antagonist eplerenone significantly decreased podocyte injury and proteinuria in rodent models of hypertensive glomerulosclerosis [68,74].

The mechanisms by which aldosterone induces proteinuria are likely complex. However, the observation that podocytes express MR suggests a direct role of liganded MR in podocyte injury. In podocytes, aldosterone treatment induces nuclear translocation of MR, activation of reduced nicotinamide adenine dinucleotide phosphate (NADPH) oxidase, and accumulation of ROS. Additionally, aldosterone increases expression of an oxidative stress effector kinase, Sgk1, and significantly delays wound- healing and promotes apoptosis in cultured mouse podocytes [69]. Taken together, these data suggest that some of the aldosterone-induced proteinuric effects and podocyte damage are derived from increases in oxidative stress in podocytes. Consistent with this hypothesis, the antioxidant tempol markly attenuates podocyte injury and proteinuria in aldosterone-infused rats and other rodent models of glomerulosclerosis. In addition to podocytes, MR is expressed in vascular endothelial and smooth muscle cells in the glomeruli, suggesting that MR action in these cells may also play a role in glomerular podocyte injury. Taken together, MR and its agonists are critical mediators of podocyte injury under several pathological conditions.

## GR (glucocorticoid receptor)

Glucocorticoid therapy is a mainstay treatment option for many forms of nephrotic syndrome. However, the mechanisms by which the glucocorticoids as GR ligands ameliorate proteinuria and inflammation-associated glomerular disease are not completely understood.

All cell types in the glomerulus express GRs [102]. Both glucocorticoid inactivating enzyme (11β-hydroxysteroid dehydrogenase type II) and GR are expressed in podocytes [103]. Moreover, cultured podocytes also express the key components of the GR-mediated signaling pathway including HSP90 and the immunophilins FKBP51 and FKBP52 [103]. Data, including our unpublished results, demonstrate that the glucocorticoid dexamethasone treatment alters gene expression patterns of cultured podocytes following either short-term high-dose treatment or long-term low-dose treatment [103]. These observations indicate that podocytes are a major target cell type for the action of glucocorticoids.

Several mechanisms accounting for the renoprotective of GR ligands effect include: 1) protecting podocytes from endoplasmic reticulum stress and rescuing a nephrin trafficking defect due to decreased N-linked glycosylation in the endoplasmic reticulum [60], 2) restoring or protecting cytoskeletal architecture by up-regulating nephrin expression [65,66], 3) inhibiting puromycin aminonucleoside (PAN)-induced reduction of phosphorylated Erk2 [67] and 4) suppressing expression of NF-κB-dependent cytokines such as IL-6 and IL-8 [65] by transrepression of NF-κB. While the GR ligands possess several renoprotective effects in glomeruli, steroid-resistance and systemic toxicity remain major issues for their long-term use. A better understanding of the mechanisms by which GR and the glucocorticoids control gene regulatory network and their crosstalk with other signaling pathways in podocytes will ensure optimal therapeutic benefits of steroid treatment.

## ER (estrogen receptor)

Podocytes express both ERα and ERβ [61]. Estrogens are protective against podocyte injuries *in vitro* and *in vivo*. First, it has long been noted that women have a better prognosis in some chronic kidney disorders than men, suggesting that gender-specific hormones play a role in glomerular development and related diseases. Second, in animal models, estrogen shows beneficial effects and protects podocytes from injury in a model of spontaneous progressive kidney disease [62] and type 2 diabetes [63]. Third, ERα knockout mice are more susceptible to the development of glomerulosclerosis and show reduced expression of desmin and nephrin [64]. Furthermore, ERα mediates estrogen protective effects from PAN-induced podocyte apoptosis both *in vitro* and *in vivo *[61]. Finally, it was recently demonstrated that ERα-mediated protective effects are associated with stabilization of mitochondrial membrane potential and activation of mitogen activated protein kinase 12 (MAPK) [61]. As ER emerges as a critical signaling molecule in the podocytes, more findings on its roles in protecting podocytes will likely surface; especially regarding its roles controlling transcriptional regulatory networks and mitogenic effects.

## Class II NRs

Class II NRs bind to HREs with direct repeats (DRs) and heterodimerize with the common partner, RXR. Class II NRs include the TRs, LXRs, RARs, RXRs, VDR, PPARs (Table 1). These NRs were considered orphan receptors until the discovery of their physiological ligands in the early 1990s. In fact, the anti-diabetic thiazolidinediones were in clinical use long before their mechanism of action as high affinity PPARγ ligands was identified [79].

## PPARs

There are three subtypes of PPAR (α, β/δ and γ) in both humans and rodents [104]. All three PPARs are expressed in the kidney and are known to play important roles in renal pathophysiology [105,106]. These receptors are major sensors for fibrates, polyunsaturated fatty acids and 15d-prostaglandin J2 and are involved in lipid metabolism and fatty acid oxidation in many tissues.

Several reports have indicated that agonists of PPARα, PPARβ/δ and PPARγ alleviate renal damage associated with ischemia/reperfusion injury in rats [75,76,80,81,107]. This renoprotective activity is associated with maintenance of nephrin and pro-survival gene expression. In another model, the anthracycline antibiotic drug doxorubicin exhibits cytotoxic effects on several organs including kidney. Doxorubicin induces renal injury by causing effacement of podocyte foot processes. Treatment of these mice with the PPARα ligand fenofibrate partially alleviates these nephritic symptoms through restoration of nephrin expression and protecting podocytes from apoptosis [77]. Predictably, PPARα knockout mice exhibit accelerated doxorubicin-induced kidney injury, diabetic nephropathy and severe proteinuria [77]. In fact, PPARα activation in podocytes resulted in an increase in nephrin expression by stimulating nephrin promoter activity, stabilizing nephrin mRNA and blocking apoptotic signaling [58,78]. PPAR ligands potently inhibit expression of proinflammatory cytokines including vascular cell adhesion molecule (VCAM-1) and IL-6 expression in various cell types [108,109] and reduce lipopolysaccharide (LPS)-induced activation of NF-κB in a PPARγ–dependent pathway in human kidney-2 (HK-2) cells [110]. Intriguingly, an increase in PPARγ expression has been observed in both rat and human kidney sclerotic conditions *in vivo*, suggesting a compensatory regulatory role of PPARγ in response to podocyte injury. *In vitro* data has also indicated that PPARγ activation protects against PAN-induced apoptosis and necrosis of podocytes [82]. In summary, the ability of PPAR agonists to protect or rescue podocytes from injury is attributed to their ability to enhance expression of slit diaphragm components such as nephrin and inhibit pro-inflammatory genes. The fact that PPARs and their ligands play key roles in lipid and cholesterol metabolism and fatty acid oxidation suggest a link between their ability to protect podocytes from injury and these metabolites and podocyte development and pathology. This will help rationalize the use of PPAR ligands in the treatment of podcoyte diseases.

## RARs

The retinoids, major ligands of the RARs, are well known developmental morphogens, important for cell specification and pattern formation in the development of many organs including limbs, lung, and kidney [111]. Vitamin A deficiency and mutations of RARs cause abnormalities in fetal kidneys, indicating that vitamin A and its receptors are essential for normal kidney development [83]. Similarly, RARα-deficient mice exhibit abnormalities in fetal kidney development and a reduced number of nephrons, the major structural and functional unit of the kidney [84].

In multiple experimental models of kidney diseases including PAN-induced nephropathy, mesangioproliferative glomerulonephritis, lupus nephritis, and diabetic nephropathy all-trans retinoic acid, the vitamin A active metabolite, has been shown to be renoprotective. All-trans retinoic acid alleviates PAN-induced proteinuria and the effacement of podocyte foot processes. In addition, administration of all-trans retinoic acid prior to PAN treatment protects animals from proteinuria and podocyte injury [85]. Consistent with this observation, recovery from PAN-induced nephropathy is significantly delayed in animals fed with a vitamin A-deficient diet [59,85]. In streptozotocin-induced diabetic and anti-Thy1.1 antibody-induced nephritis rats, retinoic acid markedly protected animals from proteinuria and renal injury. Furthermore, activation of RARs by selective ligands prevents oxidative stress-induced apoptosis in podocytes and mesangial cells [86].

In a unique class of renal disease characterized by podocyte dedifferentiation and proliferation, HIV-associated nephropathy and the collapsing glomerulopathies, the expression of retinol dehydrogenase type 1 and 9, two key enzymes in retinoic acid biosynthesis, and the overall enzymatic activity for retinoic acid synthesis were markedly reduced, suggesting that endogenous retinoic acid synthesis is impaired in diseased kidneys [87]. In a mouse model of HIV-associated nephropathy, Am580 and BD4, water-soluble RARα-specific agonists, protected animals from proteinuria, glomerosclerosis, and podocyte proliferation, and restored podocyte differentiation markers [88,89]. This is consistent with data from the knockout mouse of RARα, which leads to more aggressive kidney disease. Indeed, retinoids are known to inhibit glomerular proliferation and ameliorate glomerular lesions and proteinuria in established models of renal damage [39]. Activated RARα signaling slows the progression of kidney disease possibly by preserving the quiescent, highly differentiated state of the podocyte. As such, specific activation of the RARα can be considered a promising therapeutic strategy for patients with renal diseases associated with abnormal proliferation and cellular dedifferentiation.

As mentioned earlier, proteinruia in diabetic and non-diabetic diseases is associated with reduced podocyte expression of nephrin and podocin. All-trans retinoic acid protected podocytes from injury and enhanced nephrin and podocin expression *in vitro* and *in vivo *[90,112]. The promoter of the human and mouse nephrin gene (NPHS1) contains three putative retinoic acid response elements (RAREs) [59,66] and all-trans retinoic acid enhances nephrin promoter activity in a dose-dependent manner. However, there is no evidence demonstrating that RARs or RXRs directly target the putative RAREs in primary or cultured podocytes. *In vitro*, all-trans retinoic acid inhibits HIV-induced podocyte proliferation and restores podocyte differentiation markers. Although the exact mechanisms underlying the ability of all-trans retinoic acid to inhibit apoptosis remain unclear, suppression of a cell death pathway mediated by JNK and activator protein −1 (AP-1) has been proposed [85]. In cultured podocytes, high glucose rapidly up-regulates the monocyte chemoattractant peptide (MCP-1) mRNA transcript and protein release; however, treatment with all-trans retinoic acid suppresses MCP-1 transcription, and significantly inhibits high glucose-induced MCP-1 protein synthesis [113]. Another study demonstrated that retinoids slow down progression of renal disease by suppressing important mediators such as angiotensin II, endothelin and TGF-β in an anti-Thy1.1 nephritis rat model [114]. Moreover, recent studies have indicated that retinoids suppress NF-κB and AP-1 in non-diabetic nephropathy [115,116]. Thus, RARs may have additional renoprotective functions through the transcriptional control of podocyte-specific proteins and pro-inflammatory cytokines that are know to further escalate pathogenic cascades in the kidney.

Because all-trans retinoic acid is renoprotective in animal studies, a phase II clinical study has been approved for the use of all-trans retinoic acid to treat patients with steroid-resistant minimal change diseases including focal segmental glomerulosclerosis and collapsing glomerulopathy (ClinicalTrials.gov Identifier NCT00098020). Therefore, a complete understanding of the mechanisms underlying ATRA- and RAR-mediated renoprotective activity will further support the use of all-trans retinoic acid and the development of additional RAR agonists for the treatment of kidney disorders.

## VDR

Vitamin D deficiency is associated with proteinuria [117] and is commonly found in type 1 and type 2 diabetic patients [117-122]. These observations suggest that decreased vitamin D3 may contribute to increased risk of diabetes complications and mortality and imply an intrinsic anti-proteinuric activity for vitamin D. In fact, treatment with doxercalciferol, a VDR agonist, alleviates proteinuria and glomerulosclerosis in type 1 and type 2 diabetic animals [121,123] and prevents diet-induced obesity and insulin resistance. VDR agonists also protect animals in non-diabetic models of renal disease, including Heyman Nephritis [124], an anti-thy-1 model of mesangial proliferative glomerulonephritis [125-127], subtotal nephrectomy-associated podocyte loss and hypertrophy [128,129], PAN- or adriamycin-induced podocyte apoptosis and loss [130,131], unilateral ureteral obstruction [91] and X-linked Fabry disease-associated proteinuric renal injury [132].

Similar to ERα and RARα knockout mice, VDR knockout mice are more susceptible to diabetic kidney injury [92,133] and streptozotocin-induced diabetic kidney disease. VDR is expressed in glomerular podocytes, as well as other cell types in the glomerulus and in the tubular epithelium [134]. Using 14C-labelled vitamin D3, it was found that 1,25-(OH)2-vitamin D3 localizes in the nucleus of podocytes, suggesting a regulatory action of the VDR and vitamin D3 in these cells [134]. Treatment of diet-induced obese mice with the VDR agonist, paricalcitol, has been shown to decrease proteinuria, and podocyte injury. The same agonist also reduces proteinuria in diabetic nephropathy [132], in part by interrupting the damaged pathway initiated by lysoglobotriaosylceraminde in podocytes.

VDR appears to be highly inducible in podocytes [93], suggesting that podocytes are a main target of vitamin D3. Renal injury is accompanied by significant up-regulation of β-catenin, predominantly in podocytes and tubular epithelial cells. The VDR agonist, paricalcitol, induces a physical interaction between the VDR and β-catenin in podocytes, thereby suppressing of β*-*catenin-mediated gene transcription. Other studies have suggested that vitamin D3 elicits its anti-apototic and pro-survival response in podocytes through suppression of caspase-3 activity, TGF-β1 signaling and the expression of several apoptosis related proteins (Fas, FADD and Bax). Vitamin D3 also increases anti-apoptotic protein expression and activates bone morphogenetic protein 7 (BMP-7) signaling. The ability of vitamin D3 to block high glucose-induced angiotensinogen through inactivation of NF-κB activity has also been proposed [135]. In addition, 1,25(OH)2D3 and its analogs also induce nephrin mRNA and protein expression [66,136]. A mechanism by which 1,25(OH)2D3 induces association of VDR with its response elements in the nephrin promoter and the recruitment of RNA polymerase II and histone H4 acetylation has been proposed [94]. Similar observations have shown that 1,25(OH)2D3 reverses high glucose-induced nephrin reduction in podocytes and prevents nephrin decline in both type 1 and 2 diabetic mice [95].

In summary, a body of evidence indicates that VDR and its agonists are capable of eliciting renoprotective effects through enhancing/maintaining nephrin expression and inhibiting injurious pathways to podocytes. This suggests that VDR ligands may be promising therapeutic agents to prevent/ameliorate glomerulopathy.

## Concluding remarks

NRs control many aspects of cell differentiation, animal development and homeostasis. Some NR ligands have proven beneficial in treating human diseases including cancer, inflammatory disease and metabolic syndrome. However, our understanding of NR function in podocytes is still at an early stage, in part, due to lack of knowledge of their target genes. We know how NR functions in other tissues and cell types and how they crosstalk with other signaling pathways. For example, in addition to ligand-dependent activation of their target genes, NRs such as GR, PPAR, VDR and RAR possess ligand-dependent transrepression activity that inhibits NF-κB and AP-1 [42], and liganded PPARs and TR have been shown to crosstalk with mTOR signaling [137,138]. Are these mechanisms conserved in podocytes? Given the facts that TNFα and IL-1β, both of which are NF-κB activating agents, decrease nephrin expression, this question is highly relevant to NR action in podocytes. Are there cell type-specific functions of NRs present in podocytes? Studies on conditional and podocyte-specific knockout animals of NRs will likely provide a better understanding of the role of a given NR in podocyte development. Currently, only a handful of NRs has been studied in podocytes. It is certain that NRs not discussed in this review will also have important roles in podocyte development and related disorders. Lastly, cell-cell interactions between podocytes and glomerular endothelial cells are also critical to podocyte development and maintenance of the filtration barrier integrity. As such, NR function in glomerular endothelial cells will be equally important to explore.

## Abbreviations

AP-1: Activator protein −1; ATRA: All-trans retinoic acid; ACTN4: Alpha actinin 4; BMP-7: Bone morphogenetic protein 7; CBP: cAMP response element-binding protein; CD2AP: CD2 associated protein CD2AP; DR: Direct repeats DR; ERα/β: Estrogen receptor alpha/beta; FKBP: FK506 Binding Protein; FSGS: Focal segmental glomerulosclerosis; GRα: Glucocorticoid receptor alpha; HSP90: Heat shock protein 90; HAT: Histone acetylase; HDAC: Histone deacetylase; HRE: Hormone response binding element; HK-2: Human kidney-2; INF2: Inverted forming-2; IR: Inverted repeats; LPS: Lipopolysaccharide; LXR: Liver X receptor; mTOR: Mammlian target of rapamycin; MR: Mineralocorticoid receptor; MAPK: Mitogen activated protein kinase; MCP-1: Monocyte chemoattractant peptide; NF-kB: Nuclear factor kappa B; NR: Nuclear receptor; NCoR: Nuclear receptor corepressor; PCAF: p300/CBP-associated factor; PPAR/α/β/γ: Peroxisome proliferator-activated receptor; PR: Progesterone receptor; PAN: Puromycin aminonucleoside; RARα: Retinoic acid receptor alpha; RARE: Retinoic acid response element; RXR: Retinoid X receptor; sgk1: Serine/threonine-protein kinase 1; SMRT: Silencing mediator for retinoid and thyroid hormone receptors; SRC: Steroid receptor coactivator; TR: Thyroid hormone receptor; TGF-β: Transforming growth factor β; VCAM: Vascular cell adhesion molecule; VDR: Vitamin D3 receptor.

## Competing interests

The authors declare that they have no competing interest.

## Authors’ contributions

SK and HYK drafted and LB edited the manuscript text, figures and tables. All the authors read and approved the final manuscript.
